# Metabolic and histopathological effects of sleeve gastrectomy and gastric plication: an experimental rodent model

**DOI:** 10.3402/fnr.v60.30888

**Published:** 2016-04-15

**Authors:** Osman Bilgin Gulcicek, Kamil Ozdogan, Ali Solmaz, Hakan Yigitbas, Serdar Altınay, Aysegul Gunes, Duygu Sultan Celik, Erkan Yavuz, Atilla Celik, Fatih Celebi

**Affiliations:** 1 General Surgery Clinic, Bagcilar Training and Research Hospital, Istanbul, Turkey; 2 Pathology Clinic, Bagcilar Training and Research Hospital, Istanbul, Turkey; 3 Biochemistry Clinic, Bagcilar Training and Research Hospital, Istanbul, Turkey; 4 Veterinary Medicine, Bagcilar Training and Research Hospital, Istanbul, Turkey

**Keywords:** obesity, sleeve gastrectomy, gastric plication

## Abstract

**Introduction:**

Obesity has recently become a major health problem, and researchers have been directed to work toward the development of surgical techniques, with new mediators playing an important role in nutrition. Gastric plication (GP) and sleeve gastrectomy (SG) have become popular recently. These are widely used techniques in bariatric surgery.

**Objectives:**

In this study, we aimed to compare the efficiency of SG and GP techniques on rats.

**Methods:**

Wistar-Hannover rats (*n*=18) were divided into three equal groups, namely SG, GP, and control. Blood samples were taken before the operation and on the 30th day after the operation. The weights of all rats were recorded both on first day and the 30th day after the operation. Serum gastrin, ghrelin, and leptin levels were also measured on the same days. For histopathological examination, gastrectomy was performed after the animals were sacrificed.

**Results:**

Average weight loss was 10% for the SG group and 6.5% for the GP group. One month after the operations, the decrease in the ghrelin and leptin levels of GP and SG groups was significant compared with the levels of the control group. Gastrin levels of the SG group increased significantly compared with those of the control group. Histopathological examination revealed that there was significant decrease in the ghrelin and leptin levels of the GP and SG groups compared with those of the control group. Foveolar hyperplasia (FH), cystic glandular dilatation, and fibrosis were significantly higher in the GP and SG groups compared with the control group.

**Conclusion:**

Although GP is not as effective as SG in terms of weight loss, it provides the same effectiveness in decreasing ghrelin and leptin levels. Histopathological findings revealed that FH, fibrosis, and the cystic glandular dilatation development rates were similar.

Obesity is defined as the accumulation of abnormal or excessive fat tissue which impairs health, resulting in cardiovascular system diseases, diabetes mellitus, metabolic syndrome, musculoskeletal disorders, and psychiatric problems. Furthermore, obesity is recognized as a major health problem today (1, 2).

It has been suggested that obesity is a major threat to humanity, an assertion that has been seconded by the World Health Organization (3). Patients with a body mass index (BMI) of over 40 kg/m^2^ are considered morbidly obese (4).

Diet, changes in habits, and medical treatment are non-surgical modalities to treat obesity, although with limited effect on weight loss. As such, there is no effective, long-term treatment for obesity (5). However, surgery is considered to be effective in managing obesity, and sleeve gastrectomy (SG), gastric bypass, gastric banding, and gastric plication (GP) are the most common procedures (6). There are many studies in the literature indicating that SG and GP involve simpler techniques, fewer complications, and higher efficiency compared with other techniques (7).

In this study, we aimed to compare the metabolic efficiency and histopathological results of both restrictive surgical methods: SG and GP.

## Materials and methods

### Experimental design

Eighteen Wistar-Hannover rats (600–700 g), provided by the Bagcilar Training and Research Hospital Animal Center (BADABEM), were used in this study. All animals were housed in cages (six rats in each cage) under controlled room temperature 22(±2°C), humidity (60–70%), with 12-h light–dark schedule. They were fed with pellets, ad libitum, containing 21% protein (MBD Animal Feed, Kocaeli, Turkey). All experimental procedures were approved by the Bagcilar Training and Research Hospital Animal Care and Use Committee (2014/02).

### Study groups

Rats were grouped randomly as group 1 (control group, *n*=6), group 2 (SG group, *n*=6), and group 3 (GP group, *n*=6). All rats were sacrificed using high-dose ketamine after 30 days of operation. Blood samples from all animals were taken by intracardiac puncture, and biochemical parameters (gastrin, ghrelin, and leptin) were measured. After midline laparotomy, a total gastrectomy was done, and the specimens were fixed in 10% formaldehyde for histopathological examination.

### Operative procedure

The abdominal part of all rats was shaved under Isoflurane (Baxter, Puerto Rico, USA) inhalation anesthesia (5% for induction and 1–2% for maintenance), following the provision of antisepsis by 10% povidone iodine solution (Betadine^®^). The initial weight of all rats was measured and recorded. Blood samples (1 ml) were taken from an internal jugular vein for the biochemical parameters (gastrin, ghrelin, and leptin).

**Fig. 1:**
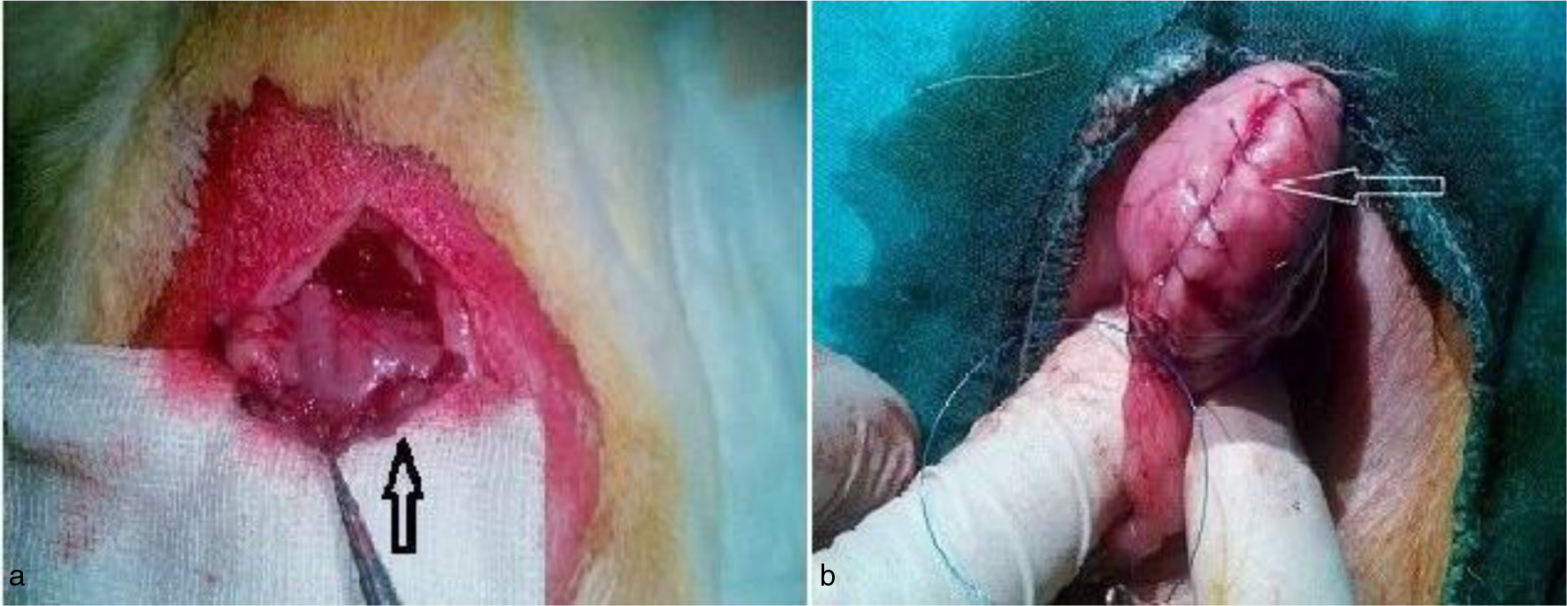
(a) Sleeve gastrectomy. The arrow shows the gastrectomy line. (b) Gastric plication. The arrow shows the plication line.

A 3-cm midline incision was done under sterile conditions. The abdomen was closed after exploration of the stomach in the control group. Fifty-to-seventy percent of the stomachs were excised after the dissection of gastrocolic and gastrosplenic ligaments from just 0.5 cm proximal to the pylorus to diaphragmatic crus in the SG group. Then, the inner layer was closed by continuous 5/0 polypropylene sutures (Dogsan, Trabzon, Turkey), and the outer layer was closed with continuous extramucosal sutures by 5/0 polypropylene (Fig. 1a). The same steps, up to the resection, were also performed on the GP group. After dissection of the ligaments, plication of the greater curvature was done with two-layered continuous extramucosal sutures by 5/0 polypropylene (Dogsan) (Fig. 1b).

All surgical procedures were performed by the same surgeon. The abdominal incisions of all rats were closed by single layered non-continuous 2/0 silk sutures (Dogsan). Thirty days after the operation, all rats were sacrificed using high-dose ketamine anesthesia (ketamine hydrochloride 50 mg/kg; Ketalar^®^, Parke-Davis, İstanbul, Turkey, and xylazine 10 mg/kg; Rompun^®^, Bayer, İstanbul, Turkey).

### Biochemical analysis

Ghrelin, gastrin, and leptin levels were measured before and 30 days after the operations. Samples and the kit (Eastbiopharme Co., Ltd., Hangzhou, China) were brought to room temperature. First, 24 ng/ml stock was obtained by standard serial dilution from 6 standard kit. Standards (50 µl) were added on the antibody-coated micro-plate wells. Then 50-µl streptavidin-HRP was placed on thumbnail standards. After pipetting of standards on a micro-plate, serum samples were pipetted as 40 µl on each micro-well. Leptin antibody, ghrelin antibody, and gastrin antibody of 10-µl and 50-µl streptavidin-HRP were added on serum samples. The micro-plates were covered and left at 37°C incubation for 60 min. After preparing the washing-solution 30×, ELISA plates were washed five times in 350 µl of washer. Fifty microliters of Chromogenic Solution A and Chromogenic Solution B were pipetted in each micro-well. Incubation was done at 37°C for 10 min in dark. Fifty microliters of Stop Solution were added on each micro-well. Micro-plate absorbance was measured at 450 nm for 10 min.

### Histological analysis

The resected specimens of the rats were fixed for pathologic evaluation in 10% buffered formaldehyde solution for one day. All of the distal portions of the material containing the glandular tissue were sampled. The tissue parts were embedded in paraffin blocks and 5-µm-thick paraffin sections were obtained for hematoxylin–eosin and Masson's trichrome stain. Microscopic examination of all tissues was done in Bagcilar Training and Research Hospital Pathology Laboratory. Foveolar hyperplasia (FH) and glandular cystic dilatation (CGD) were examined in the fundus, corpus, and antral part of the specimens. Scoring was done as semi-quantitatively (0=nope, 1=mild, 2=moderate, and 3=severe). Fibrosis of the tissue was scored (0=nope, 1=mild, 2=moderate, and 3=severe).

### Immunohistochemical evaluation

Anti-ghrelin, anti-leptin, and anti-gastrin antibodies (Rabbit polyclonal IgG-abcam, Heidelberg, Germany) were administered on lysine-coated slides for the immunohistochemical examination of the tissues. All the tissues were studied as a positive control. Cells showing immunoreactivity were counted in the fundus, corpus, and antrum of the stomach. The immunoreactive cells were calculated in five different most intensely stained regions and the average was calculated by dividing it by five under low magnification. The average value was obtained in this way. All tables of immunohistochemical analysis of all rats were done by counting the immunoreactive cells.

### Statistical analysis

In this study, statistical analysis was done by NCSS (Number Cruncher Statistical System) 2007 statistical software program (Utah, Heidelberg, USA). A Kruskal–Wallis test and descriptive statistical methods (mean, standard deviation) were used for the evaluation of data and comparison between groups, Dunn's multiple comparison test was used for subgroup comparison, and a Chi-square test was used for the comparison of qualitative data. A *p*-value of <0.05 was considered statistically significant.

## Results

Postoperative gastrin values of the SG group had increased significantly (*p*=0.011). However, there was no significant change in gastrin values post-operatively in the GP group (*p*=0.964) (Table 1).

**Table 1:** Gastrin, ghrelin, and leptin levels

		Control group	SG group	GP group	*p*
Gastrin (ng/ml)	Preop	39.47±3.34	39.98±4.5	42.32±4.55	0.469
	Postop	38.26±2.49	43.16±2.68	42.36±4.27	**0.043**
Ghrelin (ng/ml)	Preop	12.81±0.69	16.73±1.25	13.05±1.83	**0.001**
	Postop	12.97±1.42	13.29±1.20	11.47±1.58	0.092
Leptin (ng/ml)	Preop	5.03±0.86	5.52±0.76	4.41±0.41	**0.048**
	Postop	5.05±0.75	4.14±0.30	4.11±0.81	**0.008**

GP: gastric plication; Postop: 30 days after the operation; Preop: before operation; SG: sleeve gastrectomy. Bold values are statistically significant.

Ghrelin and leptin levels decreased in both groups (ghrelin/leptin SG group: *p*=0.001/*p*=0.002, GP group: *p*=0.006/*p*=0.624). However, the decrease in leptin values was not significant (*p*=0.624) (Table 1 and Graphics 1 and 2).

Weight loss was 10% in the SG group and 6.3% in the GP group (*p*=0.016 for SG and *p*=0.068 for GP group) (Table 2).

**Table 2:** Average weight of rats before and 30 days after the operation

	Control group	SG group	GP group	*p*
Preop (gr weight)	648.83±48.26	633.83±40.65	581.50±28.97	**0.027**
Postop (gr weight)	628±42.40	560.33±32.86	544.17±62.40	**0.018**

GP: gastric plication; Postop: 30 days after the operation, Preop: before operation, SG: sleeve gastrectomy. Bold values are statistically significant.

**Fig. 2:**
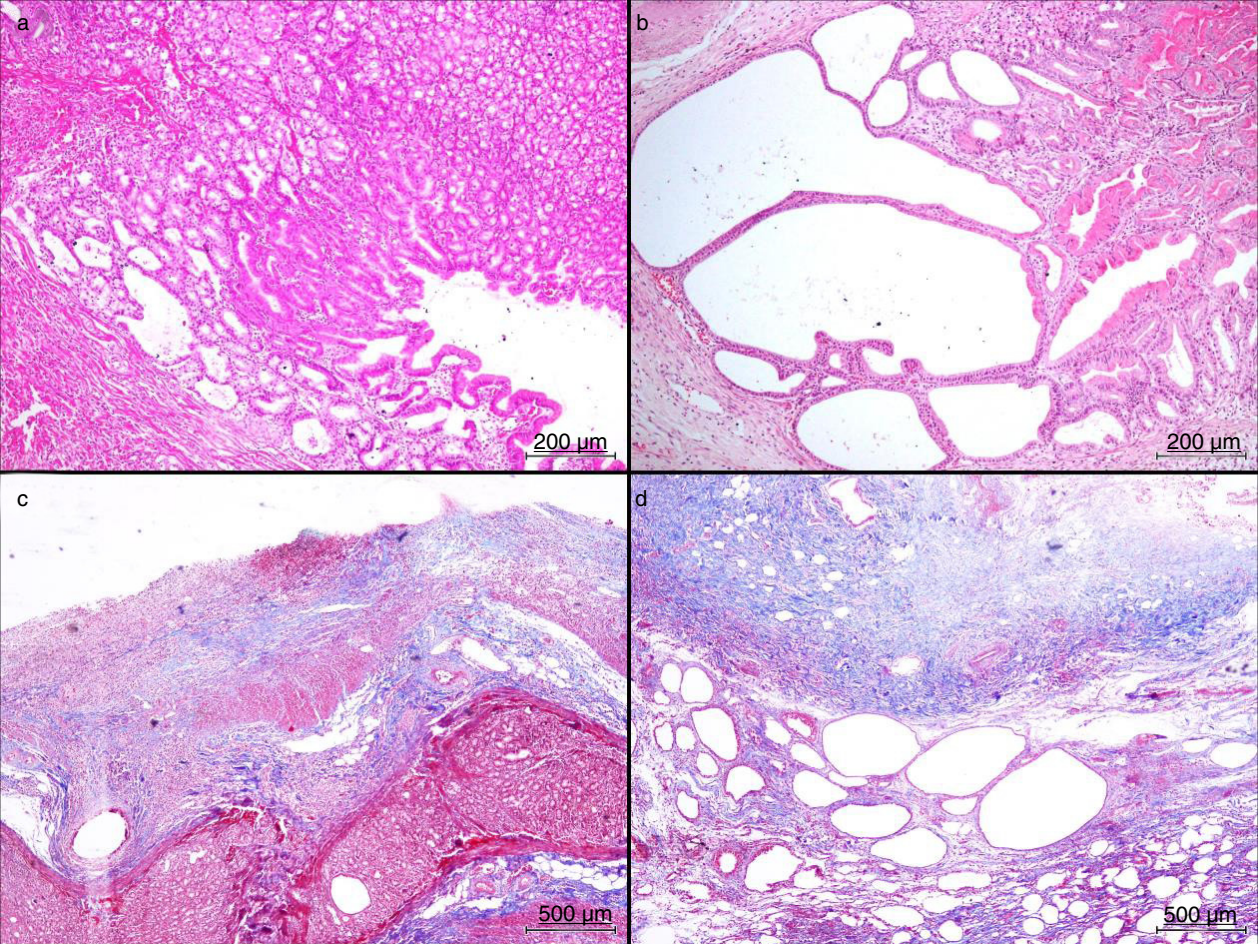
There is prominent foveolar hyperplasia (a, H&E, ×100) and also marked gastric cystic dilatation in the SG group (b, H&E, ×100) on histopathological examination. We see mild fibrosis on the serosal surface of the GP group (c, Masson's trichrome stain, ×40). Contrarily, there is prominent fibrosis on the serosal surface of the GP group, which manifest as blue colored areas on histochemical examination (d, Masson's trichrome stain, ×40) (GP: gastric plication, SG: sleeve gastrectomy).

FH, cystic glandular dysplasia (CGD), and the grade of fibrosis were significantly higher in both groups than in the control group (*p*=0.008, *p*=0.001, *p*=0.001). Although this increase was higher in the SG group, there was no significant difference between the groups (*p*>0.05) (Table 3 and Fig. 2).

**Table 3:** Distribution of FH, CGD, and fibrosis among the groups

		Control group (%)	SG group (%)	GP group (%)	*p*
FH	Minimal	6 (100.00)	1 (16.70)	1 (16.70)	**0.008**
	Mild	0 (0.00)	1 (16.70)	4 (66.70)	
	Moderate	0 (0.00)	2 (33.30)	1 (16.70)	
	Severe	0 (0.00)	2 (33.30)	0 (0.00)	
CGD	Minimal	6 (100.00)	1 (16.70)	0 (0.00)	**0.001**
	Mild	0 (0.00)	1 (16.70)	5 (83.30)	
	Moderate	0 (0.00)	2 (33.30)	1 (16.70)	
	Severe	0 (0.00)	2 (33.30)	0 (0.00)	
Fibrosis	Minimal	6 (100.00)	0 (0.00)	0 (0.00)	**0.0001**
	Mild	0 (0.00)	0 (0.00)	4 (66.70)	
	Moderate	0 (0.00)	3 (50.00)	2 (33.30)	
	Severe	0 (0.00)	3 (50.00)	0 (0.00)	

CGD: cystic glandular dysplasia, FH: foveolar hyperplasia, GP: gastric plication, SG: sleeve gastrectomy. Bold values are statistically significant.

The immunohistochemical examination revealed that antral gastrin values were significantly higher in the SG group compared with the control and GP groups (*p*=0.027, *p*=0.004). However, there was no significant difference between the control group and the GP group (*p*=0.646).

**Fig. 3:**
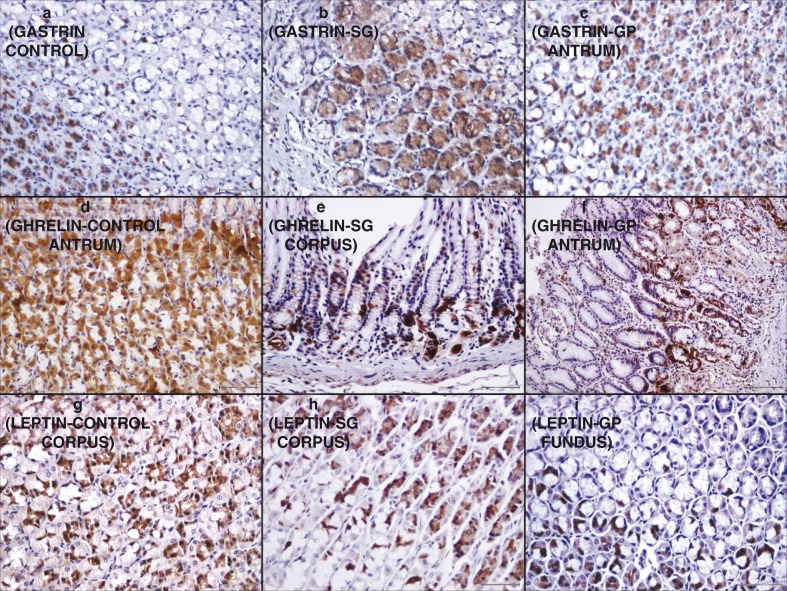
Gastrin immunoreactivity created by gastrin dye is more evident in the SG group than the GP and control groups on immunohistochemical examination (a–c, ×400). There is less ghrelin immunoreactivity in both the SG and GP groups compared with the control group (d, e, ×400; f, 200). Similarly, there is less leptin immunoreactivity in both the SG and GP groups compared with the control group (g–i, ×400) (GP: gastric plication, SG: sleeve gastrectomy).

The ghrelin and leptin levels of the SG and GP groups were significantly lower than those of the control group (ghrelin/leptin: SG group: *p*=0.0001/*p*=0.0001, GP group: *p*=0.0001/*p*=0.0001) (Table 4 and Fig. 3).

**Table 4:** Gastrin, ghrelin, and leptin values of the groups

	Control group	SG group	GP group	*p*
Gastrin antrum	54.17±2.79	60.5±2.35	55.67±3.39	**0.004**
Ghrelin fundus	51.5±5.47	25.33±5.32	28±5.62	**0.0001**
Ghrelin corpus	40.83±4.79	21.5±4.76	22.5±3.62	**0.0001**
Ghrelin antrum	31.33±4.72	18.33±3.88	20.33±4.18	**0.0001**
Leptin fundus	42.17±3.37	28±4.86	28.67±4.13	**0.0001**
Leptin corpus	22.17±3.49	16.83±2.93	18.83±2.48	**0.024**

GP: gastric plication; SG: sleeve gastrectomy. Bold values are statistically significant.

## Discussion

Our study aimed to compare the metabolic efficiency and histopathological results of both restrictive surgical methods, SG and GP. Obesity is considered to be a major health issue all over the world. It can be defined as the accumulation of abnormal or excessive fat tissue in the body that negatively impacts health. Patients with a BMI of 40 kg/m^2^ or more are considered morbidly obese (1, 4).

**Graphic 1:**
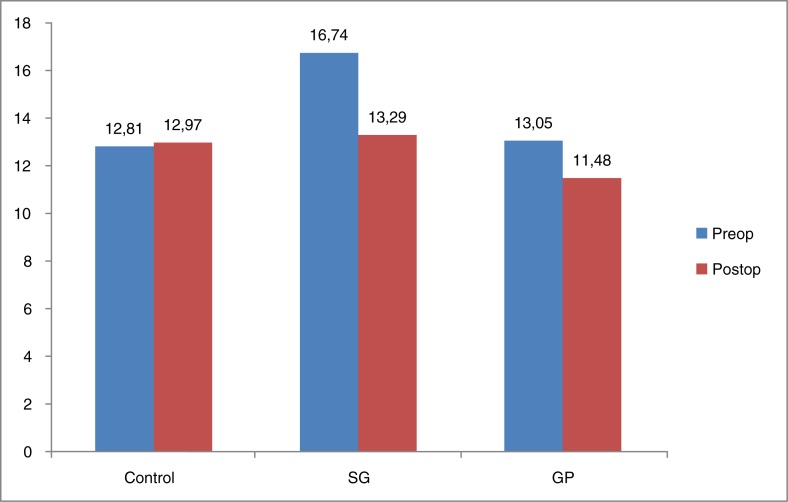
Ghrelin levels (ng/ml). SG: sleeve gastrectomy, GP: gastric plication.

**Graphic 2:**
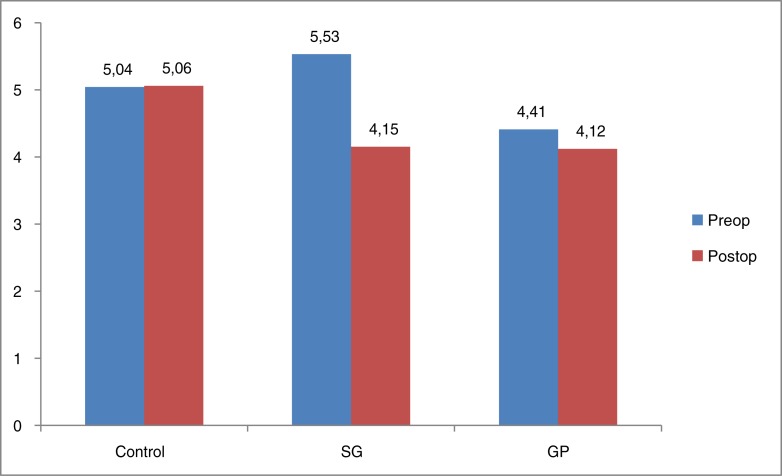
Leptin levels (ng/ml). SG: sleeve gastrectomy, GP: gastric plication.

Besides diet, changes in dietary habits, drugs, and surgical methods provide long and effective treatment for the management of obesity (5). Maklin et al. (8) reported that surgery is advantageous in terms of cost-effectiveness in dealing with obesity and its morbidities.

SG and GP have been performed with gradually increasing frequency in recent years. Both techniques can be successfully applied laparoscopically (9). SG, first described by Marceau et al. (10) in 1993, was initially applied as a first phase of biliopancreatic diversion, duodenal switch, and gastric bypass operations. Since 2003, it has been applied alone. It involves resection of the greater curvature of the stomach from antrum to angle of His throughout latarjet nerve (10).

GP was first described by Taleppour and Amoli in 2007. In this technique, greater curvature of the stomach is plicated over itself by non-absorbable sutures with the help of a 32–36 F plug catheter, after the liberalization of the greater curvature (11).

Most of the gastrin is produced and secreted by antral G cells of the stomach. The most important function of the gastrin is the stimulation of gastric acid from parietal cells of the fundus (12). All studies have demonstrated that the gastrin levels of the patients do not change or decrease after bypass procedures, except the jejunoileal bypass (13). In our study, the gastrin levels of the SG group were increased significantly (*p*=0.011), but there was no significant change in the GP group (*p*=0.964).

Ghrelin was discovered by Davis and described by Kojima in 1991 (14, 15). It is expressed by specialized enterochromaffin cells located in the fundal region of the stomach (16). Whang and Liu published an article in 2008, claiming that there is a proportional relation between BMI and ghrelin decrease (17). Leptin is produced and secreted into blood from adipocytes (18). The serum leptin level is directly proportional to the amount of body fat and is increased in obese patients (19). It has been found that the plasma leptin level is reduced in people who lose weight (20). According to the study by Considine et al., (21) 10% of weight loss causes a 53% decrease in leptin level, and the leptin levels increase again up to 70% of initial values after a 4-week period of weight loss. In our study, the ghrelin and leptin values of both groups (SG and GP) were significantly reduced after surgery, according to the biochemical and histopathological findings.

Although FH can be seen in all types of gastritis, it is more prominent in chemical gastritis (22). There is a debate about whether FH is a precursor lesion of the hyperplastic polyps (23–25). There is no malignant degeneration in FH, so it is important to distinguish these lesions from hyperplastic lesions (26). Orlowska followed 483 patients with hyperplastic polyps and 268 patients with FH for 2 years and 8 months, and detected 2% focal carcinoma in the hyperplastic polyp group. On the contrary, there was no carcinoma in the FH group (26). CGD occurs due to hyperplasia and cystic dilatation of submucosal glands extending into the submucosal layer (27). Although it is accepted as a benign lesion, there is still debate about its malignancy potential (28–31). This relation between CGD and malignancy indicates that a common factor causes both pathologies (32). In our study, FH, CGD, and fibrosis levels were significantly higher in both the SG and GP groups than in the control group (*p*=0.008, *p*=0.001, *p*=0.001). However, the SG group has higher values compared with the GP group. This may be due to the gastric resection in the SG group.

## Conclusion

Although GP does not cause weight loss as much as SG, similar metabolic activity may be provided due to the decrease in ghrelin and leptin levels after the operation. Histopathological findings revealed that SG and GP have similar results in terms of fibrosis levels, FH, and CGD formation.
